# Comparison of the MetricVBT App and the Vitruve Linear Position Transducer for Assessing Execution Velocity and ROM

**DOI:** 10.3390/jfmk11020197

**Published:** 2026-05-16

**Authors:** Tommaso Grossi, Lorenzo Micheli, Matteo Magnoni, Vahid Shoaei, Piero Benelli, Carlo Ferri Marini, Francesco Lucertini

**Affiliations:** 1 Department of Biomolecular Sciences—Division of Exercise and Health Sciences, University of Urbino Carlo Bo, 61029 Urbino, Italy; lorenzo.micheli@uniurb.it (L.M.); m.magnoni2@campus.uniurb.it (M.M.); vahid.shoaei@uniurb.it (V.S.); piero.benelli@uniurb.it (P.B.); 2 Department of Human Movement Sciences, University of Groningen, University Medical Center Groningen, 9713 Groningen, The Netherlands; c.ferri.marini@umcg.nl

**Keywords:** smartphone application, load–velocity profile, velocity-based training, strength and conditioning

## Abstract

**Background**: The primary aim of this study is to evaluate the concurrent validity and practical applicability of the MetricVBT smartphone application compared with the Vitruve linear position transducer (VitruveLPT) for measuring mean velocity (MV) and peak velocity (PV) at one-repetition maximum (1-RM) during the Smith machine bench press (SMBP). A secondary aim is to assess the range of motion (ROM). **Methods**: Eighteen resistance-trained men completed a single 1-RM SMBP exercise test, with barbell kinematics simultaneously recorded using VitruveLPT and MetricVBT. Between-device differences were assessed using Wilcoxon signed-rank and paired-sample *t*-tests with Bonferroni correction (α ≤ 0.05). Associations were examined using Spearman’s (ρ) and Pearson’s (r) correlations, and absolute agreement was evaluated via intraclass correlation coefficients (ICC) and Bland-Altman analyses. **Results**: Significant differences were observed for MV (*p* = 0.026), but not for PV (*p* = 0.143) or ROM (*p* = 0.130). PV showed a very high correlation (r = 0.91, *p* < 0.001), whereas MV (ρ = 0.65, *p* = 0.002) and ROM (ρ = 0.55, *p* = 0.018) demonstrated moderate correlations. Agreement was good for PV (ICC = 0.888), moderate for MV (ICC = 0.612), and poor for ROM (ICC = 0.236). Mean bias was small for MV (−0.02 m·s^−1^) and PV (0.02 m·s^−1^), whereas ROM showed a larger bias (1.64 cm) and wide limits-of-agreement (LoA) for all variables (MV: −0.07 to 0.04 m·s^−1^; PV: −0.08 to 0.11 m·s^−1^; ROM: −13.82 to 17.10 cm). **Conclusions**: Although no statistically significant differences were observed, MetricVBT did not meet the reliability criteria for velocity monitoring. Despite small mean bias, the wide LoA for MV, PV, and ROM indicates that MetricVBT and VitruveLPT are not interchangeable for assessing performance parameters.

## 1. Introduction

Resistance training (RT) intensity is traditionally prescribed as a percentage of one-repetition maximum (1-RM), which is defined as the maximum amount of load that a given subject can lift successfully in one repetition [1]. However, the main limitation of prescribing RT based on fixed 1-RM percentages (%1-RM) relies on its inability to account for daily fluctuations in maximal strength caused by factors such as fatigue, food and fluid intake, sleep quality, and training adaptations [2,3]. Consequently, fixed %1-RM may not accurately reflect the athlete’s current strength level, and the number of repetitions that can be completed at the same relative load may vary substantially between individuals [4,5]. For this reason, several alternative approaches, such as autoregulation methods, have been proposed over the years, including repetitions in reserve and velocity-based training (VBT) [6]. It has been broadly demonstrated that barbell velocity, particularly mean velocity (MV), mean propulsive velocity (MPV), and peak velocity (PV) which are commonly used in VBT to guide load prescription during RT sessions, are strongly correlated with %1-RM (e.g., MV: R^2^ = 0.979, MPV: R^2^ = 0.981, and PV: R^2^ = 0.960) [7,8], and can influence performance outcomes and perceived exertion [9]. In this context, VBT, which uses lifting velocity to prescribe and adjust RT intensity and volume [10,11], represents a valuable approach. VBT is based on the strong inverse linear relationship [12] between relative load and lifting velocity that allows the construction of a load–velocity profile (LVP), which provides useful information about the athlete’s neuromuscular status and readiness [7,13,14].

Another important aspect of the LVP is the possibility of estimating the 1-RM load without performing a direct maximal test, which has been shown to be highly fatiguing, stressful, and time-consuming [15,16]. Given the inverse linear relationship between load and movement velocity, the velocity associated with the 1-RM load, commonly referred to as the minimum velocity threshold, can be used to estimate maximal strength [17]. Thus, exercise professionals can predict 1-RM load without performing a direct maximal test, supporting strength monitoring and individualized load prescription.

However, its effectiveness also depends on the criterion validity and test–retest reliability of the devices used to measure movement velocity [18]. Although linear position transducers demonstrate superior criterion validity compared with other technologies (e.g., accelerometers and optical systems) [19,20], their cost and expertise in their uses may limit accessibility in applied settings [21].

To address these limitations, more affordable and user-friendly solutions have emerged over the years [22]. Among these, the MetricVBT smartphone application [23] has been developed and validated for iOS or iPad OS devices [24]. The MetricVBT smartphone application, unlike LPTs, which directly measure displacement through cable extension, analyzes video footage using a computer vision system and a repetition-detection algorithm to provide two-dimensional (2D) real-time key performance metrics such as range of motion (ROM), MV, and PV during RT.

Generally, smartphone-based systems represent a low-cost and accessible alternative to traditional measurement technologies, even though their accuracy may be influenced by technical factors such as camera positioning, barbell orientation, and the type of movement [25]. Additional limitations may include barbell tilt, camera distortion, and movement in the coronal plane, as well as missed repetitions (i.e., performed repetitions not detected by the system) and ghost repetitions (i.e., false repetitions detected despite not being performed) [26]. These sources of error may be particularly relevant at the low velocities typically reached during 1-RM attempts and short displacement phases, where small tracking inaccuracies may affect velocity and ROM estimates.

In this regard, Trowell et al. [27] compared MetricVBT with a Vicon system for free-weight back squat (from 25 to 100% 1-RM) and free-weight bench press (from 50% to 100% 1-RM). Although the ROM showed a substantial agreement in squat (CCC: 0.958–0.975) and moderate in bench press (i.e., CCC: 0.901–0.926), MV was systematically overestimated in the squat (0.09–0.13 m·s^−1^) and bench press (0.06–0.09 m·s^−1^), with proportional bias observed, suggesting limited accuracy for VBT prescription. Such errors may compromise VBT prescription by overestimating true movement velocity, thereby affecting load selection, training-zone classification, and longitudinal performance monitoring [28]. However, it is worth noting that the aforementioned validation was performed across multiple submaximal and maximal loads and under free-weight conditions, which typically involve higher movement velocities and fewer mechanical constraints compared with 1-RM testing. Therefore, device validity may be load-dependent, as measurement accuracy under submaximal conditions may not necessarily translate to the very low velocities and different demands associated with 1-RM load. Moreover, that study did not consider PV (i.e., the instantaneous velocity reached during the concentric phase), a fundamental velocity variable commonly used for ballistic exercise prescription (e.g., bench press throw) [29] which provides complementary information regarding explosive force production that is not fully captured by MV or MPV metrics. The PV is usually associated with strength and power-related performance outcomes and is directly reported by MetricVBT, making its validity practically relevant for coaches and practitioners who may use the application across different RT contexts and exercises [30]. Furthermore, another validation proposed by Šagovac and Baković [31] primarily examined moderate loading ranges (i.e., 45–60–75% of 1-RM load); thereby, evidence is lacking under heavy-load conditions, which are critical for accurate 1-RM load estimation and minimal velocity threshold assessment [32] and controlled bar-path exercises such as the Smith machine (SMBP).

To date, no studies have examined MetricVBT’s validity and applicability during the SMBP, a controlled and linear movement frequently used to assess upper-body strength and establish LVPs. Therefore, the primary aim of this study is to determine the concurrent validity, between-device agreement, and practical applicability of the MetricVBT smartphone application for measuring MV and PV at 1-RM load during the SMBP, using the Vitruve linear position transducer (VitruveLPT) as a comparison device. As a secondary aim, the same analysis was extended to ROM.

## 2. Materials and Methods

### 2.1. Participants

A total of 18 resistance-trained men (see Table S1, Supplementary Digital Content [SDC]) with no previous experience in VBT were recruited for this study. The inclusion criteria were: (*i*) a minimum of 1 year of RT experience, aligned with the National Strength and Conditioning Association RT status classification [33], with the average participant classified as having an intermediate experience level; and (*ii*) no history of upper limb injuries. All the participants obtained medical clearance to perform maximal strength exercises before being enrolled. Each participant was asked to maintain their habitual diet and physical activity and avoid vigorous exercise in the 24 h before the testing session, as well as caffeine and alcohol on testing days. All participants were informed about the research scope and procedure before signing a written informed consent form before participation. The study was approved by the Institutional Review Board of the School of Sport Sciences of the University of Urbino (Italy) and conducted in the spirit of the Declaration of Helsinki.

### 2.2. Study Design

For this inter-device comparison study, participants completed a single experimental testing session. During the experimental session, participants were first familiarized with SMBP procedures. In particular, each participant was instructed to perform the SMBP exercise and the corrected SMBP execution was supervised and verified by an experienced operator to ensure: (*a*) shoulder blades were retracted and in contact with the bench along with the pelvis; (*b*) feet were placed solid on the ground; (*c*) a prone grip with hands at 150% of the acromion–acromion distance was used [34]; (*d*) the barbell was lifted from the chest to full elbows extension; and (*e*) the barbell was lowered under control until touching the meso-sternal point [35] and held until the operator signals to avoid the rebound effect. During the propulsive phase, participants were instructed to lift the barbell at maximal intended velocity, with verbal encouragement and velocity feedback provided for each repetition [36]. Subsequently, in the same session, the participants performed an experimental trial using the velocity-based LVP protocol proposed by Sanchez-Medina et al. [37].

### 2.3. Procedures

Before the test, subjects performed a 5 min warm-up of stationary cycling at 100 W followed by 5 min of upper-body joint mobilization exercises. After that, each participant accomplished the LVP velocity-based protocol [37]. The LVP velocity-based protocol begins with: (*a*) an initial set of 5 repetitions at 20 kg; and (*b*) progressive incremental sets, with the barbell load increased by 10 kg until MPV was < 0.5 m·s^−1^; thereafter, the barbell load was increased by 5 kg if the lift was successful or decreased by 2.5 kg if the lift was unsuccessful. The 1-RM load was directly assessed and considered the highest amount of load lifted with full elbow extension. Rest intervals were 2–3 min for the light (i.e., MPV > 1.0 m·s^−1^) and medium (i.e., 0.65 m·s^−1^ ≤ MPV ≤ 1.0 m·s^−1^) loads, with 3 and 2 repetitions performed for light and medium loads, respectively. For the heaviest loads (i.e., MPV < 0.65 m·s^−1^), five to six minutes of rest were provided. A maximum of five attempts was allowed to determine the 1-RM.

### 2.4. Measurement Equipment

Body height and body mass (Seca, Hamburg, Germany) were evaluated during the familiarization session, while all sessions were performed on a Smith machine (Technogym, Cesena, Italy). Barbell kinematics were simultaneously recorded using two linear position transducers (Vitruve, Madrid, Spain; 1000 Hz) and the MetricVBT smartphone application (Core Advantage Pty Ltd., Oakleigh South, Australia).

The transducers were placed perpendicular to the barbell and attached to its left side. This setting was adopted because the Vitruve Teams software (version 1.30.10) did not allow simultaneous display of MV and MPV outputs, treating both as one selectable variable, not as two independent simultaneous outputs in the workflow. Barbell MV was calculated as the vertical displacement of the barbell divided by the duration of the concentric phase. PV was defined as the highest instantaneous velocity reached during the concentric phase. Barbell ROM was determined as the vertical displacement of the barbell from the chest mesosternal point (i.e., bottom position) to the end of the concentric phase (i.e., top position) of each repetition. Only MV, PV, and ROM values corresponding to the 1-RM load were included in the analyses. The MPV was only recorded since the velocity-based LVP protocol adjusts its load increments according to the MPV values. However, given the incompatibility of the MetricVBT smartphone application to record the MPV, it was excluded from the statistical analysis.

**Figure 1:**
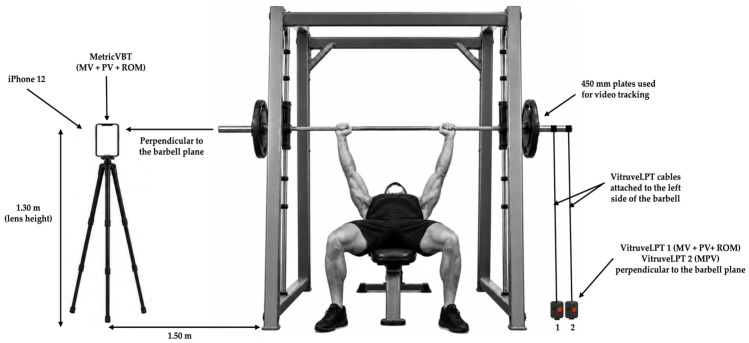
Experimental setup: the MetricVBT app was operated on an iPhone 12 mounted on a tripod positioned 1.50 m laterally to the rack, with the lens set at 1.30 m height and oriented perpendicular to the barbell plane to record mean velocity (MV), peak velocity (PV), and range of motion (ROM). Two Vitruve linear position transducers (VitruveLPT) were placed on the floor on the opposite side of the rack, directly perpendicular to the barbell’s right side, to simultaneously record MV, PV, ROM, and mean propulsive velocity (MPV). This graphical illustration was generated with AI assistance (ChatGPT, version 5.5) and subsequently edited by the authors to accurately represent the experimental setup.

The MetricVBT smartphone application, running on an iPhone 12 (Apple Inc., Cupertino, CA, USA), captured barbell displacement via video tracking of 450 mm plates during the concentric phase. The application records high-definition 720p video at 60 frames·s^−1^ with automatic tracking corrections. The iPhone was mounted on a tripod placed 1.50 m to the right of the rack, with lens at 1.30 m height and perpendicular to the barbell plane according to the manufacturing rules, ensuring both plates and the participants remained fully visible during each attempt. All testing sessions were conducted under the same indoor lighting conditions, and all videos were analyzed by the same operator to minimize inter-rater variability (see Figure 1).

### 2.5. Statistical Analyses

Statistical analyses were performed using JASP (version 0.18.1; University of Amsterdam, Amsterdam, The Netherlands). Data are presented as mean ± SD for normally distributed variables and as median (interquartile range, [IQR]) for non-normally distributed variables. Normality of differences between MetricVBT and VitruveLPT measurements was assessed on within-subject difference scores of the Shapiro–Wilk test. Non-normally distributed data were analyzed using the Wilcoxon signed-rank test; otherwise, a paired *t*-test was applied. Based on the distribution of paired difference scores, the MV and ROM were analyzed using the Wilcoxon signed-rank test, while a paired-sample *t*-test was performed for PV (α = 0.05). Effect sizes were reported as rank–biserial correlation (r_rb_) for Wilcoxon tests and Cohen’s d for paired *t*-test, each with 95% confidence intervals (CI) to quantify the magnitude of mean differences between devices; agreement was primarily assessed using intraclass correlation coefficients (ICC_2,1_) and Bland-Altman analyses. Between-device associations were examined using Pearson’s correlation coefficient (r) for PV, whereas Spearman’s rank-order correlation coefficient (ρ) was applied to MV and ROM due to the presence of outliers visually detected (see Figure 1). Specifically, MV showed two discrepant values (−0.09 m·s^−1^), attributed to MetricVBT. ROM showed three discrepant values, two related to MetricVBT (20.43 cm and 17.13 cm) and one related to VitruveLPT (−17.88 cm), reflecting recording errors (see Figure 1). The magnitude of the correlation coefficients was interpreted according to the criteria proposed by Mukaka [38], where values of 0.30–0.50 indicate low correlation, 0.50–0.70 moderate correlation, 0.70–0.90 high correlation, and ≥0.90 very high correlation. The ICC_2,1_ corresponding to a two-way random-effects model for single measurements based on absolute agreement, with 95% CI, was interpreted according to the thresholds proposed by Koo and Li [39]: poor (<0.50), moderate (0.50–0.75), good (0.75–0.90), and excellent (>0.90). Root mean square error (RMSE) was additionally calculated to quantify the overall magnitude of between-device measurement error. To quantify absolute inter-device measurement error, typical error was calculated from paired differences (SD_diff_/√2) for MV, PV, and ROM. Percentage TE (%) was additionally calculated by dividing TE by the mean of both devices and multiplying by 100. Bland-Altman analyses were performed to assess agreement between paired differences obtained from the VitruveLPT and MetricVBT systems for MV, PV, and ROM. Mean bias and 95% limits of agreement (LoA) were calculated. Proportional bias was evaluated using Pearson’s correlation coefficient (r) between the mean of the two devices and their difference scores. The assumption of homoscedasticity was assessed by visual inspection of residual and Bland-Altman plots. Homoscedasticity was observed for MV and PV, whereas ROM showed evidence of heteroscedasticity, primarily driven by influential outlying observations. Exploratory sensitivity analyses were conducted to examine whether visually identified influential observations materially affected agreement and reliability estimates. The results of this analysis are provided in the SDC.

## 3. Results

Participants’ (age: 25.10 [23.75–26.05] yrs; 1-RM load: 93.83 ± 19.63 kg; Realtive strength index: 120 ± 21%) descriptive characteristics, together with barbell velocities measured with the VitruveLPT and MetricVBT smartphone application, are presented in Table S1.

For MV (W = 29.00; *p* = 0.026; r_rb_ = –0.621, 95% CI [–0.853, –0.183]), the Wilcoxon signed-rank test indicated significant differences between devices. While for ROM (W = 121.00; *p* = 0.130; r_rb_ = 0.415, 95% CI [–0.084, 0.748]), no significant differences were found. Similarly, for PV, the paired-sample *t*-test showed no significant difference between devices (t = 1.534; df = 17; *p* = 0.143; Cohen’s *d* = 0.362; 95% CI [–0.121, 0.834]).

Regarding between-device correlations, PV exhibited a very high positive correlation (r = 0.91, 95% CI [0.767, 0.966], *p* < 0.001), whereas MV (ρ = 0.65, 95% CI [0.263, 0.857], *p* = 0.003) and ROM (ρ = 0.55, 95% CI [0.110, 0.808], *p* = 0.018) demonstrated moderate positive correlations.

Absolute agreement assessed via ICC_2,1_ was moderate for MV (ICC_2,1_ = 0.612, 95% CI [0.200, 0.837]; RMSE: 0.033 m·s^−1^), poor for ROM (ICC_2,1_ = 0.236, 95% CI [−0.240, 0.623]; RMSE: 7.896 cm), and good for PV (ICC_2,1_ = 0.888, 95% CI [0.727, 0.956]; RMSE: 0.051 m·s^−1^). Typical error was 0.020 m·s^−1^ (12.9%) for MV, 0.035 m·s^−1^ (10.0%) for PV, and 5.62 cm (14.6%) for ROM.

**Figure 2:**
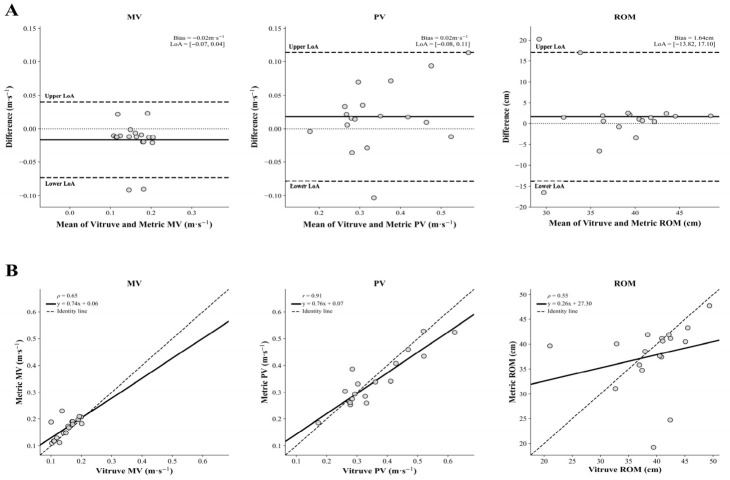
Comparison between VitruveLPT and MetricVBT during the Smith machine bench press. Panel (**A**): Bland-Altman plots showing the mean bias (solid line) and 95% limits of agreement (LoA) (dashed lines). Mean velocity (MV) values were concentrated within the low-velocity range, and two observations fell below the lower LoA. Peak velocity (PV) showed moderate scatter without an obvious systematic pattern. Range of motion (ROM) displayed markedly wider dispersion, indicating poorer agreement between devices. Panel (**B**): Correlation plots showing the regression line (solid) and the identity line (dashed); r, Pearson correlation coefficient; ρ, Spearman rank-order correlation coefficient.

Bland-Altman analyses (see Figure 2) showed a mean bias of −0.02 m·s^−1^ (95% CI: −0.03 to −0.002 m·s^−1^) for MV, with 95% LoA ranging from −0.07 m·s^−1^ (95% CI: −0.10 to −0.05 m·s^−1^) to 0.04 m·s^−1^ (95% CI: 0.02 to 0.07 m·s^−1^). For PV, the mean bias was 0.02 m·s^−1^ (95% CI: −0.01 to 0.04 m·s^−1^), with 95% LoA ranging from −0.08 m·s^−1^ (95% CI: −0.12 to −0.04 m·s^−1^) to 0.11 m·s^−1^ (95% CI: 0.07 to 0.16 m·s^−1^), representing a substantial proportion of the measured values.

For ROM, the mean bias was 1.61 cm (95% CI: −2.34 to 5.57 cm), with 95% LoA ranging from −13.98 cm (95% CI: −20.83 to −7.13 cm) to 17.21 cm (95% CI: 10.36 to 24.06 cm), reflecting very poor individual-level agreement. No evidence of proportional bias was observed for any variable (MV: r = −0.13, *p* = 0.596; PV: r = 0.39, *p* = 0.111; ROM: r = −0.12, *p* = 0.632).

## 4. Discussion

The purpose of this study is to evaluate the concurrent validity and practical applicability of the MetricVBT smartphone application compared to the VitruveLPT for measuring MV and PV during the SMBP at 1-RM load as a primary aim. Additionally, as a secondary aim, the same analysis was extended to ROM.

Regarding the primary aim, the MetricVBT differed in velocity measurements from VitruveLPT for MV (*p* = 0.026). Conversely, PV reported no significant differences between devices. Likewise, no significant differences were observed for ROM. In terms of association, MV showed a moderate correlation (ρ = 0.65, *p* = 0.003), whereas PV demonstrated a very high correlation between devices (r = 0.91, *p* < 0.001); ROM also showed a moderate positive correlation (ρ = 0.55, *p* = 0.018) [38].

Nevertheless, analyses of absolute agreement revealed moderate agreement for MV (ICC_2,1_ = 0.612, 95% CI [0.200–0.837]), poor agreement for ROM (ICC_2,1_ = 0.236, 95% CI [−0.240, 0.623]), and good agreement for PV (ICC_2,1_ = 0.888, 95% CI [0.727–0.956]). Although PV demonstrated a very high correlation, none of the observed ICC values reached the thresholds previously suggested by Courel-Ibáñez et al. [40] for velocity-monitoring devices (e.g., ICC ≥ 0.99), whereas MV and especially ROM remained below commonly accepted levels for precise interchangeable use in velocity-based training contexts.

Furthermore, Bland-Altman analyses showed trivial mean biases between devices for MV (–0.02 m·s^−1^) and PV (0.02 m·s^−1^), indicating no systematic over- or underestimation. However, the wide 95% LoA for MV (–0.07 to 0.04 m·s^−1^) and for PV (–0.08 to 0.11 m·s^−1^) indicates substantial measurement error at the individual level, suggesting that individual measurements differ between systems despite a small mean bias. For ROM, a larger mean bias (1.64 cm) and wide LoA (–13.82 to 17.10 cm) further indicate considerable between-device disagreement and limited precision for individual assessments.

Overall, these findings suggest that MetricVBT may track important training parameters without evidence of proportional bias for MV, PV, or ROM. Nonetheless, due to the differences in MV and wide LoA, its precision for exact velocities and displacement values appears limited at the individual level, indicating that MetricVBT and VitruveLPT should not be considered interchangeable.

As reported in Figure 2, two MV data points (–0.09 m·s^−1^) fell outside the LoA and were attributable to a measurement error from the MetricVBT. In addition, three ROM data points (20.43 cm, 17.13 cm, and −17.88 cm) deviated markedly beyond the negative and positive LoA, respectively. Examination of these observations at the individual level, based on the consistency of the measurements across repetitions for the same participant, suggested that two of the ROM discrepancies (17.13 and 20.43 cm) were due to incorrectly recorded values from the MetricVBT, whereas the remaining discrepancy (−17.88 cm) was incorrectly recorded from the VitruveLPT. Indeed, these values were inconsistent with the other repetitions recorded for that subject. When these erroneous observations were removed, the agreement between devices improved substantially, as shown by the sensitivity analysis (see Table S2 and Figure S1). The sensitivity analysis revealed that the LoAs improved from −0.03 to 0.01 m·s^−1^ (mean bias: −0.01 m·s^−1^) for MV and −4.26 to 5.51 cm (mean bias: −0.01 cm) for ROM, together with higher ICC values and higher correlations for all variables (i.e., MV, PV, and ROM). Therefore, from a practical standpoint, these findings suggest that the MetricVBT may potentially provide accurate performance values and may represent a valuable tool when recording errors are identified and excluded. However, the sensitivity analysis cannot be interpreted as evidence of improved device performance. Moreover, exercise professionals should standardize data-quality procedures (e.g., appropriate camera positioning, plate visibility, consistent recording conditions, and repeated measurements when feasible) to minimize tracking errors and improve measurement consistency, also considering the typical measurement error.

Notably, these discrepant values may also be attributed to the biomechanical characteristics of the sticking region typically observed during 1-RM trials [41]. Maximal bench press loads are performed at very low mean velocities (e.g., 0.16 m·s^−1^), which are highly sensitive to neuromuscular fatigue and velocity loss [28]. In this regard, in our study, lower execution velocities may have increased potential differences between devices, particularly for MV and ROM. Conversely, PV, defined as the instantaneous highest velocity achieved during the concentric phase, may be less influenced by the sticking point since the PV occurs before the sticking phase, when bar acceleration is still increasing.

Our findings are partially consistent with previous literature [42], where authors investigated the validity of the Metric smartphone application against Gymaware linear position transducer during free-weight resistance exercise (i.e., back squat, bench press, and deadlift from 45, 55, 65, 75, 85, and 100% of 1-RM). When considering the bench press, a moderate agreement for MV (CCC = 0.935) together with a systematic bias of 0.043 m·s^−1^ and a wide LoA (−0.102 to 0.188 m·s^−1^) was found. For ROM, poor agreement was reported (CCC = 0.547), with an average overestimation of 4.3 cm, wide LoA (−10.9 to 8 cm), and presence of proportional bias, indicating greater overestimation. Our findings showed only moderate agreement for MV and poor agreement for ROM with a wide LoA, despite the absence of proportional bias.

In contrast to the present findings, Šagovac and Baković [31] reported strong correlations for MV (r = 0.93), PV (r = 0.91), and ROM (r = 0.75), and excellent reliability (MV [ICC = 0.93 to 0.95, PV ICC = 0.89 to 0.97 and ROM (ICC = 0.95 to 0.97]) obtained with the MetricVBT during the submaximal free-weight bench press exercise. However, in our study, ROM showed only a moderate correlation (ρ = 0.55) and poor absolute agreement (ICC = 0.236), indicating substantially lower interchangeability between systems. This reduced agreement may be related to methodological differences, as their study examined moderate-to-high loads (i.e., 40 to 80% 1-RM) under free-weight conditions, whereas the present investigation tested maximal load (i.e., 1-RM load) and slower bar velocities in a Smith machine. They also reported a systematic overestimation of ROM (SEM = 4.38 ± 4.14 cm), whereas the present study observed a smaller mean bias (1.64 cm) but markedly wider LoA (−13.82 to 17.10 cm), reflecting greater individual-level variability.

Trowell et al. [27] also highlighted clear differences by comparing the MetricVBT and Vicon system. Specifically, moderate agreement for ROM (CCC = 0.901–0.926) with systematic underestimation of −0.74 to 1.44 cm and relatively narrow LoA (−5.80 to 3.55 cm) was observed in the free-weight bench press. Conversely, our findings showed a larger ROM bias and wider LoA, indicating greater variability between systems. Regarding MV, the same investigation showed an overestimation of 0.06−0.09 m·s^−1^ and proportional bias increasing with velocity, whereas in the present study, minimal mean bias (−0.02 m·s^−1^) and no proportional bias (r = −0.03, *p* = 0.596) were found.

Our findings agree with Taber et al. [24], who reported a moderate correlation for MV (r = 0.67) and minimal systematic bias when comparing smartphone-based tracking with a reference system. Similarly, we observed moderate MV correlation (ρ = 0.65) and trivial mean bias (−0.02 m·s^−1^), supporting the interpretation that smartphone-based systems can detect performance trends but lack precision for interchangeable measurements.

Finally, the present results are consistent with Renner et al. [26], who found larger random error for MV (RMSE = 0.04–0.14 m·s^−1^) when smartphone-based applications, including MetricVBT, were compared with a Vicon system. However, it is worth noting that none of the aforementioned studies analyzed PV. Our findings demonstrate that despite this variable reporting a good ICC value and minimal bias, according to the wide LoA, the MV, PV, and ROM cannot be accurately measured.

From a practical perspective, MetricVBT may be a promising smartphone application for tracking performance parameters and offers a simple, accessible alternative for barbell velocity monitoring. Nevertheless, significant differences in MV and the limited absolute agreement for MV, PV, and ROM suggest it cannot replace VitruveLPT for precise individual assessments or for detecting small within-athlete changes. Moreover, as highlighted in the Methods section, MetricVBT reports MV rather than MPV, likely because it uses a fixed 0.1 m·s^−1^ threshold to define repetition onset and termination. This approach may include non-propulsive phases or exclude valid effort, thereby limiting its usefulness in practical settings. MPV reflects only the propulsive phase, during which the applied force exceeds gravity and the load is actively accelerated, whereas MV also includes the braking phase. The inclusion of braking actions can weaken the load–velocity relationship and underestimate true performance, especially at light loads where the braking phase is more pronounced, making MPV particularly relevant.

Some limitations of the present study should also be considered. Our sample included only male participants, and the use of SMBP may limit the generalizability of the results to free-weight exercises involving greater bar-path variability or to other populations. No a priori sample size calculation based on expected agreement precision was performed. Therefore, CIs for both the mean bias and the LoA were reported to quantify the uncertainty surrounding the Bland-Altman analyses. The width of these CIs should be considered when interpreting the precision of the agreement analyses, particularly for variables showing wider LoA. Specifically, MV demonstrated relatively narrower LoA and CI ranges, suggesting greater precision and lower between-device variability. In contrast, PV showed wider LoA and broader CI ranges relative to the measured values, indicating lower precision of agreement estimates. ROM exhibited the widest LoA and CI ranges, reflecting substantial uncertainty and poor individual-level agreement between devices. This represents an important limitation of the present study. Accordingly, future investigations should evaluate MetricVBT’s validity across diverse exercises, intensity zones, and participant groups, ideally using gold-standard (i.e., 3D motion-capture systems) as references. Further research is warranted to investigate its validity across different exercise modalities, loading ranges, and velocity metrics (i.e., mean vs. peak values), and to determine whether ongoing improvements in video-based tracking accuracy could enhance its suitability for individualized monitoring. In addition, future studies should also examine the test–retest reliability of MetricVBT and related measurement procedures across multiple testing sessions, as the present design only allowed inter-device comparison and did not permit assessment of between-session reliability or measurement stability over time.

## 5. Conclusions

In conclusion, the MetricVBT smartphone application reported significant differences from the VitruveLPT for MV, while no differences were reported for both PV and ROM. Partial concurrent validity was observed for PV with a very high correlation, followed by MV and ROM with moderate correlations. Absolute agreement varied across variables, being good for PV, moderate for MV, and poor for ROM, with wide LoA for MV, PV, and ROM, indicating substantial individual-level measurement error.

These findings indicate that although MetricVBT demonstrated a very high to moderate association, none of these values met the reliability criteria for velocity monitoring (e.g., ICC ≥ 0.99). Additionally, despite a small mean bias, the wide LoA indicates that MetricVBT and VitruveLPT are not interchangeable for assessing performance parameters.

## Data Availability

The data presented in this study are available on request from the corresponding author.

## Supplementary Materials

The following supporting information can be downloaded at: https://www.mdpi.com/article/10.3390/jfmk11020197/s1, Table S1: Descriptive statistics of participant characteristics and barbell velocities; Table S2: Sensitivity analysis of agreement between VitruveLPT and MetricVBT after removing potential influential observations; Figure S1: Sensitivity analysis. Comparison between VitruveLPT and MetricVBT during the Smith machine bench press.
