# Supplementary material for: Comparison of the MetricVBT App and the Vitruve Linear Position Transducer for Assessing Execution Velocity and ROM
Source: J Funct Morphol Kinesiol. 2026 May 16;11(2):197. doi: 10.3390/jfmk11020197 (PMC13214824; doi:10.3390/jfmk11020197)
Supplement: Supplementary file 1 [file jfmk-11-00197-s001.zip › jfmk-4258978-supplementary.pdf]

Supplementary file

Table S1. Descriptive statistics of participant characteristics and barbell velocities measured with the VitruveLPT and MetricVBT smartphone application during the 1-RM in the Smith-machine bench press.

Variable	Mean ± SD	Median [IQR]	Shapiro-Wilk W	Shapiro-Wilk p-value
Age (yrs)	–	25.10 [23.75 - 26.05]	0.864	0.014*
Height (m)	–	1.77 [1.74 - 1.80]	0.885	0.031*
Weight (kg)	78.61 ± 9.55	–	0.969	0.786
1-RM (kg)	93.83 ± 19.63	–	0.948	0.394
Relative strength (%)	120 ± 21	–	0.981	0.962
VitruveLPT MV ($m \cdot s^{-1}$)	0.15 ± 0.03	–	0.936	0.251
MetricVBT MV ($m \cdot s^{-1}$)	0.17 ± 0.04	–	0.938	0.267
VitruveLPT PV ($m \cdot s^{-1}$)	0.36 ± 0.12	–	0.909	0.081
MetricVBT PV ($m \cdot s^{-1}$)	0.34 ± 0.10	–	0.933	0.221
VitruveLPT ROM (cm)	–	40.63 [37.42 - 41.92]	0.874	0.021*
MetricVBT ROM (cm)	–	39.55 [36.58 - 41.18]	0.845	0.007*

Values are presented as mean ± standard deviation (SD) and median interquartile range (IQR); MV, mean velocity; PV, peak velocity; 1-RM, one-repetition maximum; ROM, range of motion; Relative strength, 1-RM divided by body weight multiplied by 100; *, significant deviation from normality ($p \leq 0.05$).

Table S2. Sensitivity analysis of agreement between VitruveLPT and MetricVBT after removing potential influential observations.

Variable	n	Mean bias	95% LoA	Proportional Bias (r, p)	RMSE	ICC (95% CI)	Pearson (r, p)	Spearman (ϱ, p)	Paired Test (p)	Effect Size (95% CI)
**MV ($m·s^{-1}$)**	15	-0.009	-0.028 to 0.010	-0.001 p=0.996	0.012	0.926 (0.544 - 0.979)	0.959 (<0.001)	0.938 (<0.001)	Wilcoxon (0.016)	-0.733 (-0.911 to -0.329)
**PV ($m·s^{-1}$)**	15	0.011	-0.087 to 0.109	0.465 p=0.081	0.045	0.907 (0.773 - 0.964)	0.923 (<0.001)	0.875 (<0.001)	t-test (0.422)	0.214 (-0.302 to 0.722)
**ROM (cm)**	15	0.625	-4.256 to 5.506	0.190 p=0.497	2.269	0.817 (0.582 - 0.927)	0.821 (<0.001)	0.779 (<0.001)	Wilcoxon (0.135)	0.450 (-0.09 to 0.786)

MV, mean velocity; PV, peak velocity; ROM, range of motion; n, sample size; Mean bias, average difference between VitruveLPT and MetricVBT measurements; LoA, limits of agreement; Proportional bias, assessed using Pearson correlation coefficient (r) between the mean of the two devices and their difference scores; p, probability value; RMSE, root mean square error; ICC, intraclass correlation coefficient; CI, confidence intervals; ϱ, Spearman rank-order correlation coefficient; Effect sizes, expressed as rank-biserial correlation for Wilcoxon signed-rank tests and Cohen's d for paired t-tests.

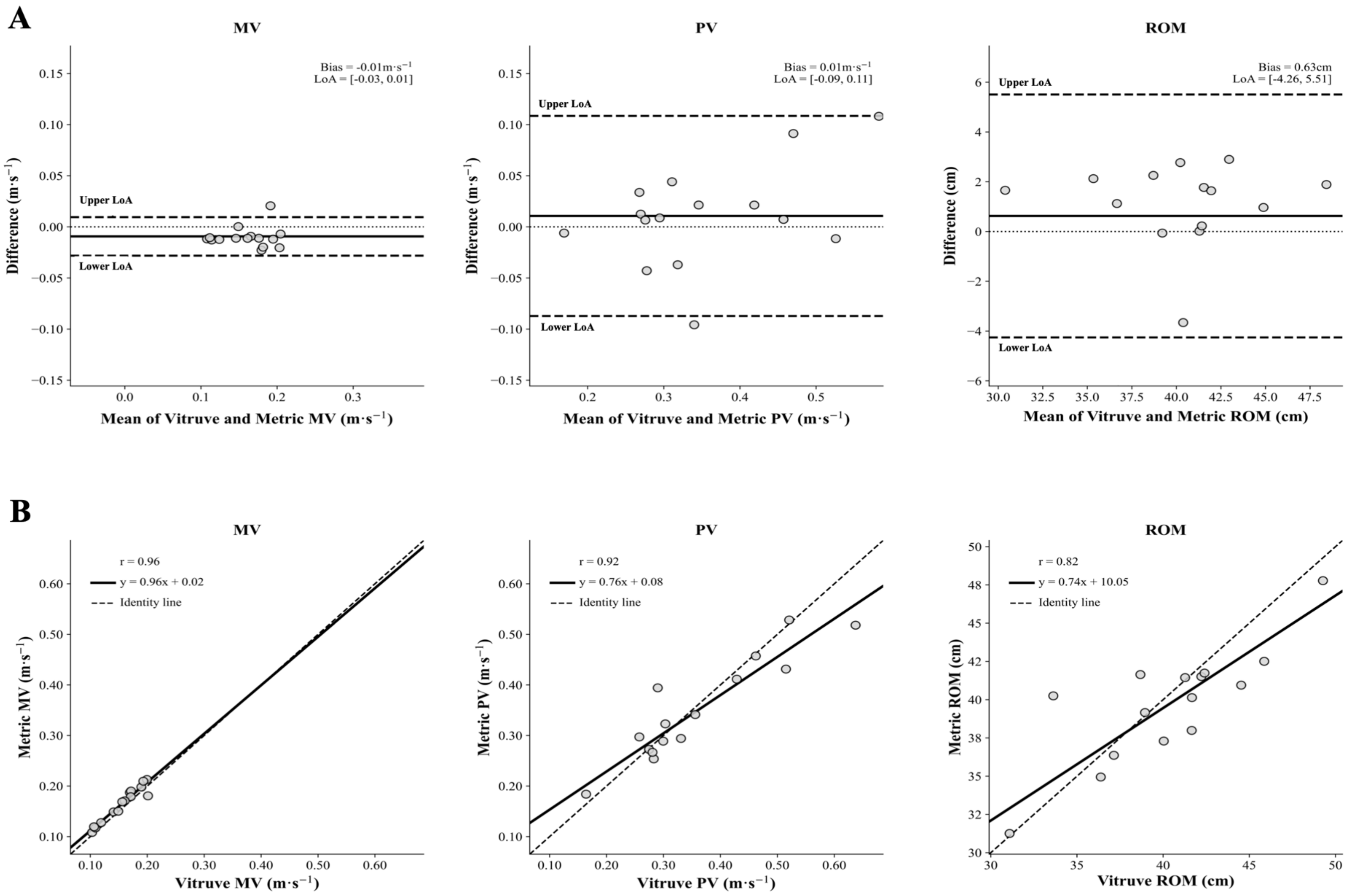

Figure S1. Sensitivity analysis. Comparison between VitruveLPT and MetricVBT during the Smith-machine bench press. Panel A: Bland-Altman plots showing the mean bias (solid line) and 95% limits of agreement (LoA) (dashed lines). Panel B: Correlation plots showing the regression line (solid) and the identity line (dashed); MV, mean velocity; PV, peak velocity; ROM, range of motion; r, Pearson correlation coefficient.
